# Maternal slow-release nitrogen diets during late gestation optimize the energy metabolism in calves’ skeletal muscle

**DOI:** 10.1371/journal.pone.0338860

**Published:** 2026-01-30

**Authors:** Thaís Correia Costa, Diana Carolina Cediel-Devia, Germán Darío Ramirez-Zamudio, Karolina Batista Nascimento, Mateus Pies Gionbelli, Marcio de Souza Duarte

**Affiliations:** 1 Department of Animal Science, Universidade Federal de Viçosa, Viçosa, Minas Gerais, Brazil; 2 Department of Animal Science, Universidade Federal de Lavras, Lavras, Minas Gerais, Brazil; 3 College of Animal Science and Food Engineering, São Paulo University, São Paulo, Brazil; 4 Department of Animal Biosciences, University of Guelph, Guelph, Ontario, Canada; UMIT TIROL Private University for Health Sciences and Technology GmbH: UMIT TIROL Private Universitat fur Gesundheitswissenschaften und -technologie GmbH, GERMANY

## Abstract

The current study aimed to determine the enriched biological process, through proteomic and transcriptome data, associated with maternal slow-release nitrogen diets received during late gestation on the skeletal muscle of the offspring. At day 180 to day 268 of gestation a total of 16 pregnant Brahman cows, were assigned into Control treatment (CON; n = 7), where cows were fed *ad libitum* a low crude protein basal diet plus mineral mixture; or Slow-released nitrogen (SRN, n = 9) based diet, where cows were fed a basal diet plus a slow-release nitrogen supplement. Muscle biopsy was performed at day 45 of age in calves and used to perform RNA sequencing (RNA-seq) and proteomic (HPLC-MS/MS) analyses. Although the experimental treatment did not show effects on transcript abundance, proteomic analysis revealed significant differences in protein expression. Enriched (adjusted *p*-value ≤ 0.05) biological processes from the exclusive proteins identified in calves’ skeletal muscle from SRN group are related to central energy metabolism (synthesis of Acetyl-CoA, tricarboxylic acid cycle, isocitrate metabolic process), regulation of calcium and nitrogen transport, and protein folding. Protein-protein interaction network assessed in the differentially abundant proteins (DAPs) revealed 4 main enriched biological processes, including ATP metabolic process, glucose metabolism, tricarboxylic acid cycle, and sarcomere organization. These findings suggest that maternal supplementation whit slow-release nitrogen during late gestation can positively influence postnatal energy metabolism in calf skeletal muscle.

## Introduction

Beef cattle productivity in extensive system depends on the interaction between basal nutrition, supplementation and genetics [1]. The availability and quality of forage-based diets vary throughout the year due to climatic factors such as rainfall, temperature and solar radiation. During the dry season, the low-quality forages are deficient in nitrogen compounds, limiting microbial enzymes synthesis required for efficient fiber degradation [2]. Nitrogen supplementation optimizes fiber degradation by alleviating nitrogen deficiency in the rumen, thereby increasing energy availability for microbial and animal growth [3]. In addition, to dietary nitrogen, microbial growth in the rumen is supported by recycled nitrogen from urea-N, which originates from absorbed nitrogen and amino acid mobilization. This mechanism can substantially contribute to ruminal nitrogen availability under low-quality forage diets [4,5].

It is well established that nutrient requirements of beef cows increase as pregnancy advances [6]. Since pregnant cows raised under extensive production are vulnerable to nutrient deficiency, including nitrogen, our research group has evaluated strategies of nitrogen supplementation on maternal and progeny performance. Protein supplementation for beef cows grazing low-quality forages at late gestation reduced maternal skeletal muscle mobilization [7]. Similar strategy used during mid-gestation preserved maternal reserves, in addition to increase glucose availability for fetal development [8]. Protein supplementation during mid or late gestation increased offspring’s rib eye area [9]. Moreover, RNA sequencing analysis revealed enhanced hypertrophic processes and overall energy metabolism in calves’ skeletal muscle [10]. In contrast, protein restriction during mid-gestation led in calves with fewer muscle fibers, higher collagen content and a transient shift toward glycolytic metabolism [11], which may negatively affect beef quality.

Based on our previous studies and recent advances in technologies that regulate nitrogen release to improve digestive and energy-nitrogen efficiency, we hypothesized that the use of slow-release nitrogen sources during bovine development can be optimized, primarily by enhancing pasture energy utilization by cows during gestation. This optimization may thereby enhance skeletal muscle development in the offspring. Thus, our objective was to integrate transcriptome and proteome data to identify the biological processes involved in the skeletal muscle development in response to maternal nitrogen optimization during gestation.

## Materials and methods

### Animal treatment and sampling

The Ethical Committee on Animal Use of the Department of Animal Science at Universidade Federal de Viçosa, Minas Gerais, Brazil (protocol 55/2022) approved all the procedures prior to the beginning of the experiment. All animals used in this study are part of the Beef herd of the Department of Animal Science of the Federal University of Viçosa, MG – Brazil, where the animal trial was performed.

Sixteen pregnant Brahman cows (579.31 ± 99.55 kg), were randomly assigned to one of two dietary treatments: Control treatment (CON; n = 7), where cows were fed *ad libitum* a low crude protein (CP) basal diet (6% CP) containing corn silage and sugarcane bagasse, plus mineral mixture (130 g/cow/d); or Slow-released Nitrogen (SRN, n = 9) based diet, where cows were fed a basal diet plus a slow-release N supplement (Timafeed Boost, Roullier Group, Saint-Malo, France). The diet’s composition is shown in Table 1. Dietary treatments were administered from day 180 (± 1.6) to day 268 (± 2.6) of gestation. The diets were provided daily as a total mixed ration, with cows fed twice a day (0700 h and 1300 h) and body weight (BW) regularly monitored to adjust the amount of supplement provided. After this period, cows were transferred to a *Brachiaria brizantha* cv. Marandu paddock and managed as a single group receiving an additional corn silage and concentrate until parturition. Cow-calf pairs from both treatments were kept in pasture under similar conditions. At 30 days of age the calves received additional supplementation through a creep-feeding system.

**Table 1:** Ingredients, level of inclusion and composition of experimental diets.

Ingredients	Inclusion (%)	
	SRN^a^	CON^b^
Slow-release nitrogen (Timafeed)^c^	27.4	–
Ground Corn	53.3	–
Wheat meal	10.0	–
Mineral mixture	9.30	–
Dicalcium phosphate	–	47.0
Sodium chloride	–	29.7
Limestone	–	16.8
Mineral Premix	–	6.50
	**Chemical composition (%)**	
DM	49.89	47.15
CP^d^	11.75	5.06
NDFap^d^	39.73	53.05
EE^d^	2.21	1.89
MM^d^	4.58	3.58

^a^SRN = supplied at the level of 2 g/kg of body weight (40% CP);^b^ CON = supplied at the level of 25 g/100 kg of body weight; DM = dry matter; CP = crude protein; NDFap = neutral detergent fiber corrected for ash and protein; EE = ether extract; MM = mineral matter; ^c^Analytical constituents: Crude ash – 40%, Insoluble ash in hydrochloric acid – 25%, Crude protein – 116%, Crude fibre – 2%, Crude oil and fats – 2%, Sodium – 3%, Magnesium – 1.5%. Additives: Urea and its derivatives - 3d1 Urea 380000 mg per kg – Binder- 1g568 Clinoptilolite of sedimentary origin 110000 mg per kg, E562 Sepiolite 63000 mg per kg – Mycotoxin Reducers- 1m558 Bentonite 95000 mg per kg. Composition: distillers’ dried grains and solubles, Concentrated distillers solubles, Sodium sulphate, Yeasts, and Ammonium sulphate. ^d^Dry matter basis.

At 45 days of age calves were submitted to a muscle biopsy sampling, and tissue was collected from the right side of *Longissimus* muscle located between the 12^th^ and 13^th^ ribs. The biopsy site was initially shaved and sanitized with 4% chlorohexidine and 70%-ethanol alcohol. Following the sanitization, the biopsy site was anesthetized with 2% lidocaine HCl (6 mL). A 2 cm incision was then created using a sterile blade 10 scalpel across the skin and subcutaneous fat (when present). Next, muscle tissue was excised by using an 8-mm biopsy punch and rinsed with sterile phosphate-buffered saline solution. Visible connective (epimysium) and subcutaneous fat tissue (when present) were trimmed off, and the muscle sample was immediately snap-frozen, powdered in liquid nitrogen, and then stored at −80°C until analysis of gene expression and protein abundance of intramuscular adipogenesis markers.

### RNAseq data generation and analyses

Muscle samples were sent to BGI Tech Solutions (Hong Kong, China). The RNA was extracted, and the library was generated through DNBSEQ Eukaryotic Strand-Specific mRNA library following the manufacturer’s instruction. The sequencing of the stranded mRNAs was performed in a DNBseq – G400 platform (MGI Tech, Shenzhen, China), following the 150 bp paired-end (PE150) protocol.

The quality control from raw data was performed by FASTQc (version 0.11.5) [12] and low-quality reads were filtered with Trimmomatic (version 0.36) [13]. The adapters, the short reads (<50pb) and low-quality reads (Phred score < 20) were removed. The remaining reads were mapped against the reference genome (*Bos taurus*, release 110) using STAR software (version 2.7.9a) [14]. Counting was performed by STAR software (version 2.7.9a) [14] while mapping was carried out using the Ensemble annotation file (release 110).

Differentially expressed (DE) genes were assessed using the limma [15] package in the R [16] environment. Genes with consistently low expression (< 100 raw counts in at least 50% of the samples within a group) were removed using the filterByExpr function. Normalization and mean–variance modeling were then carried out with voom function, which transforms raw counts into log-counts per million (logCPM) while estimating precision weights. The statistical model included the fixed effects of maternal treatments and offspring’s sex. Genes were considered DE when the adjusted *p*-value ≤ 0.05, corrected according to Benjamini-Hochberg method [17].

Isoforms were explored using the Cuffdiff tool (Cufflinks 2.2.1). The tool counts reads, normalizes transcript expression as fragments per kilobase of transcript per million mapped reads (FPKM), and models fragment counts using a beta negative binomial distribution to account for cross-replicate variability and mapping ambiguity. Input files included the mapped reads, the indexed genome (generated through STAR genomeGenerate mode), and the reference annotation file (Ensembl release 110). Cuffdiff allows the comparison of isoform-level expression between treatments and identifies isoforms that are differentially expressed or alternatively spliced. The *p*-values reported by Cufffdiff were corrected for multiple testing by using the false discovery rate (FDR) method [17], and differentially expressed (DE) isoforms were deemed significant when adjusted *p*-value ≤ 0.05.

### Shotgun proteomics data generation

Protein was extracted from 0.1 g of tissue in 1mL lysis buffer [7 M urea, 2 M thiourea, 4% CHAPS, 1% DTT and 1% protease inhibitor cocktail (Sigma-Aldrich®, St Louis, MO, USA)] homogenized using a shaft-type homogenizer (LabGEN 125, Cole-Parmer, Bunker Hill, IL, USA). The homogenate was centrifuged at 20,200 x g for 20 min at 4^o^C. The supernatant was collected, and protein quantification was estimated by Bradford protein assay (Bio-Rad, Hercules, CA, USA). Proteins were digested using a solution containing 50 mM ammonium bicarbonate (Ambic) and 20 ug trypsin (Promega, Madison, WI, USA) overnight at 37^o^C. Tryptic peptides were resuspended with trifluoroacetic acid (TFA) and desalted using Zip-Tip®C18 (Merck Millipore, Billerica, MA, USA) according to the manufacturer’s protocol. Samples were dried in SpeedVac centrifuge (AG-22331, Eppendorf, Germany) and sent to SPARC BioCentre (SickKids, Toronto, ON, Canada) for protein identification and quantification performed in a Evosep One liquid chromatography system (Evosep, Ondese C, Denmark) coupled with the Orbitrap Fusion Lumos mass spectrometer (Thermo Fisher Scientific, San Jose, CA, USA).

Raw data was processed with MaxQuant (version 2.4.7.0) [18,19] software, considering the variable (protein amino terminal acetylation, methionine oxidation) and fixed (cabamidomethylation of cysteine) modification. Label-free quantification (LFQ) mode was included, with a minimum ratio count of 1 [20]. The trypsin specificity was kept as the digestion mode and the instrument selected was Orbitrap, set to default parameters, including the parameters of first (20 ppm) and main (4.5 ppm) search peptide tolerance. The *Bos taurus* (ID = UP000009136) and *Bos indicus* (ID = UP000515132) reference proteomes were obtained from Uniprot (www.uniprot.org) database (version from October/2023).

### Functional analysis

The protein-protein interaction network of the exclusive proteins identified in SRN treatment was built with the available *Bos taurus* database using String 12.0 (string-db.org) with the default options (medium confidence = 0.40) and connections defined according to statistical evidence. The enriched biological processes of the exclusive protein were deemed significant when the adjusted *p*-value ≤ 0.05. The differentially abundant proteins (DAPs) network was built with ClueGo 2.5.7 [21], a Cytoscape (version 3.10.1) application. The enriched biological processes (BP) from *Bos taurus* database were assessed considering the right-sided unilateral hypergeometric test and *p-*values correction based on Benjamini-Hochberg method [22,23]. Only the BP identified with the adjusted *p*-value ≤ 0.05 were considered.

### Statistical analysis

Prior to analysis, proteins identified by site, reverse, and potential contaminants were filtered and removed. For the identification of the differentially abundant proteins (DAPs), only proteins occurring in at least 50% of the biological replicates of both experimental groups were kept. The peptide intensities were log2-transformed and normalized based on the robust linear regression (RLR) method. The statistical model followed the structure:


$$\gamma_{ijk}=\mu +T_{i}+S_{J}+Q_{k}+\varepsilon_{ijk}$$

Where, $\gamma_{ijk}$ is the normalized log2-transformed intensity of peptide, μ the intercept, $T_{i}$ is the fixed effects of treatments (SRN and CON), $S_{J}$ is the fixed effect of progeny’s sex (Female and Male), $Q_{k}$ is the random effect of sequence, and $\varepsilon_{ijk}$ is the random error associated with $\gamma_{ijk}$ with $\varepsilon_{ijk}\;\sim \;\text{N}\;(\text{0},\sigma_{e}^{\text{2}})$. Adding the peptide sequences as one of the random effects predictors is highly recommended as individual peptide effects are often quite strong. The contrast was made between treatments, with significance based on ANOVA considering the DAPs when *q-value* ≤ 0.05. All analyses were performed using the MSqRob [24,25] packages in R environment [16].

## Results

### Transcriptome profile

From the whole skeletal muscle transcriptome, an average of 24.1 million reads/sample was kept after the steps of quality control and trimming. An average of 84.8% of the reads were mapped against *Bos taurus* reference genome (Ensemble, release 110) (S1 Table). After mapping, a total of 14,003 genes were identified, whereas 964 and 1,330 genes were found exclusively in the treatments SRN and CON, respectively (S1 Fig). These genes were not considered differentially expressed and may reflect low-abundance transcripts or sequencing noise rather than biologically meaningful differences. A multidimensional scaling (MDS) plot was performed for all genes expressed in all samples and showed no clear separation between treatments (S2 Fig), demonstrating that our experimental treatment did not reflect in transcriptome alterations. Indeed, the heat map from the 100 most variable genes did not cluster the treatments due to the divergences in terms of gene expression (S3 Fig).

The analysis of differentially expressed genes was performed with a total of 11,709 genes present in both treatments (intercept, S1 Fig). Adopting the pre-established adjusted *p*-value ≤ 0.05 there were no up or downregulated genes (S4 Fig), reinforcing the lack of difference between treatment at transcriptional level.

### Proteomic profile

**Fig 1:**
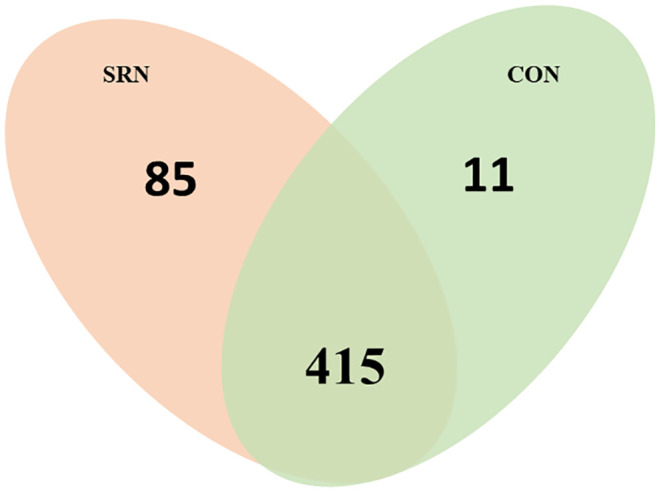
Venn diagram of the proteins identified. Number of proteins identified in each treatment (exclusive), the intercept containing the number of proteins common in both treatments. SRN = Slow-released Nitrogen; CON = Control.

A total of 756 proteins were identified. After the exclusion criteria, a total of 511 proteins were maintained, whereas 85 and 11 proteins were found exclusively in treatment SRN and CON, respectively. Controlling the *q*- value ≤ 0.05, a total of 27 proteins were differentially abundant (DAPs) between groups, in which 25 proteins were more abundant in the SRN group. Only two DAPs were more abundant in CON group (Fig 1). The list of DAPs is shown in Table 2.

**Table 2:** Differentially abundant proteins (DAPs) in the skeletal muscle of the calves.

Accession^a^	Protein name	Gene name^b^	*q-*value	log2(FC)^c^ (SRN vs CON)
A0A6P5CY36	Acetyltransferase component of pyruvate dehydrogenase complex	**DLAT**	0.021	−0.719
A0A6P5BTN2; A0A6P5BTN9	Citrate synthase	**CS**	0.049	−0.689
A0A6P5CD73; A0A6P5CFI7	Pyruvate kinase	**PKM**	0.029	0.312
A0A6P5D8Y3	Myosin-binding protein C, fast-type	MYBPC2	< 0.01	0.333
A0A6P5C027	non-specific serine/threonine protein kinase	OBSCN	0.049	0.358
A0A6P5AWY4	Myomesin-2	**MYOM2**	< 0.01	0.365
A0A6P5C4U2	Voltage-dependent anion-selective channel protein 1	VDAC1	0.048	0.377
A0A6P5BU16	Myosin-binding protein C, slow-type	**MYBPC1**	< 0.01	0.398
A0A6P5CJC9	Titin	TTN	< 0.01	0.425
A0A6P5BTL4	ATP synthase subunit beta	**ATP5B**	0.016	0.432
A0A6P5B758	F-actin-capping protein subunit alpha	CAPZA2	0.041	0.436
A0A6P5B2U0; A0A6P5BHT6	Phosphoglucomutase-1 isoform X1	**PGM1**	0.015	0.437
A0A6P5DBL6	Creatine kinase M-type	CKM	0.012	0.446
A0A6P5DY27	Fructose-bisphosphate aldolase	**ALDOA**	0.012	0.455
A0A6P5BKK6;A0A6P5BR02; A0A6P5BR29	Filamin-C isoform X1, X2, X3	**FLNC**	< 0.01	0.462
A0A6P5BIB0	Alpha-actinin-2	ACTN2	< 0.01	0.467
A0A6P5DXI6	Sarcoplasmic/endoplasmic reticulum calcium ATPase 1	ATP2A1	0.009	0.480
A0A6P5DVI2	ATP synthase subunit alpha	**ATP5A1**	0.012	0.482
A0A6P5E276	Malate dehydrogenase	**MDH2**	0.012	0.572
A0A6P5BH70	Voltage-dependent anion-selective channel protein 3	VDAC3	0.043	0.589
A0A6P5CCD0	Myosin-7	LOC109564202	0.012	0.591
A0A6P5DCX1	Myosin-2	LOC109573380	< 0.01	0.614
A0A6P5BVQ0	Galectin	LGALS1	0.049	0.673
A0A6P5BSD8	Triosephosphate isomerase	**TPI1**	< 0.01	0.681
A0A6P5DCX1; A0A6P5DCX9	Myosin-2, Myosin-3	LOC109573380, LOC109573382	0.009	0.818
A0A6P5BJP7; A0A6P5BJQ9; A0A6P5BJQ3; A0A6P5AU49; A0A6P5AU58	Tropomyosin alpha-3 chain isoform X1, X3, X2, X4, X5	TPM3	0.012	1.333
A0A6P5CQY6	ATP synthase subunit gamma	**ATP5C1**	0.022	3.977

SRN = Slow-release nitrogen; CON = Control; ^a^Proteins with more than one accession number represent different isoforms or variants; ^b^Gene names in bold means they were associated with significant biological processes (Fig 3); ^c^Negative and positive log2(fold change) indicate lower and greater abundance in treatment SRN compared to CON, respectively.

**Fig 2:**
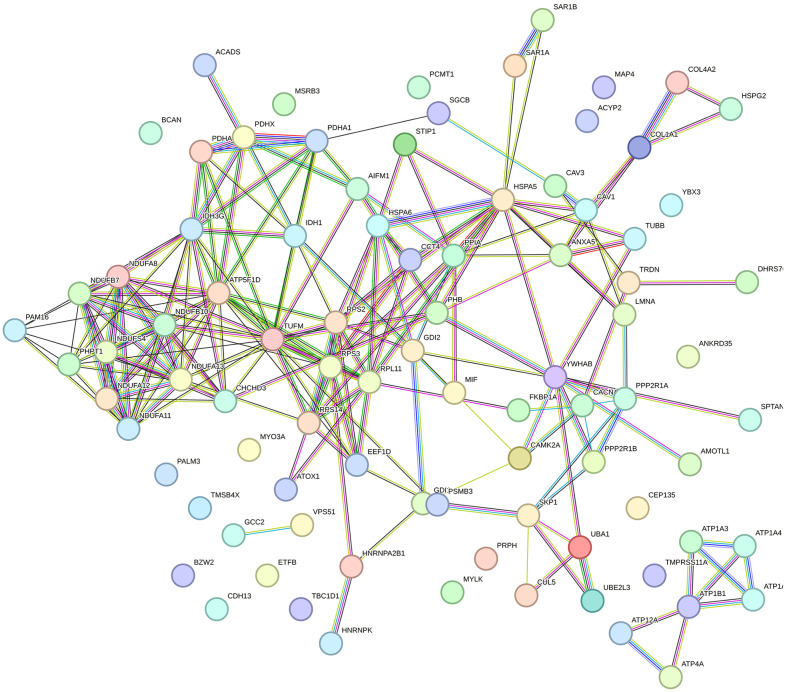
Protein interaction network of the exclusive proteins in the skeletal muscle of calves from slow-release nitrogen (SRN) treatment. Nodes represent the differentially abundant proteins, and the lines represent the connections between proteins.

**Fig 3:**
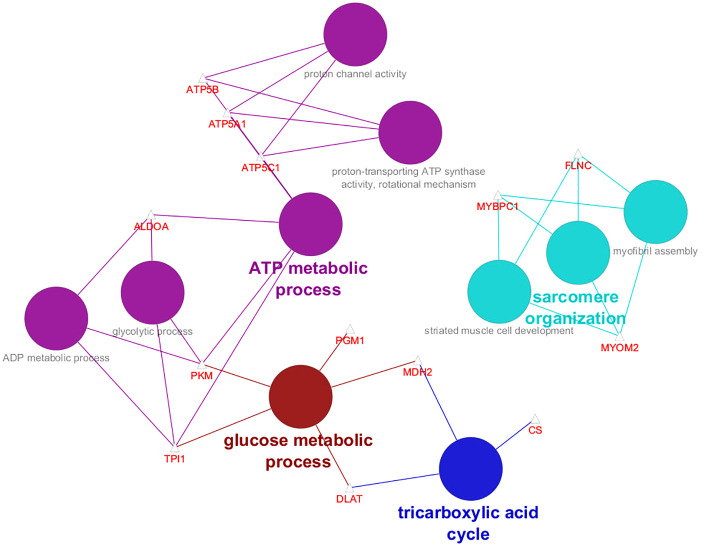
Analyses of the enriched biological process of the differentially abundant proteins (DAPs). Network connecting the DAPs (triangles) with the enriched biological processes (circles). The node colors represent the functional group.

### Function of the exclusive proteins identified in the skeletal muscle of calves

The interaction network of the exclusive proteins identified in SRN (Fig 2) was significant (adjusted *p*-value < 1.0 × 10 ⁻ ¹⁶), indicating non-random functional associations and suggesting that the set of proteins is at least partially biologically connected as a group. In contrast, the interaction network of the exclusive proteins from CON was not significant (adjusted *p*-value = 0.197), indicating a random collection of proteins with limited functional connectivity. Therefore, only the enriched biological process of the exclusive proteins in SRN treatment will be further discussed. Table 3 shows the biological process of interest influenced by the exclusive proteins identified in SRN treatment. The enriched biological processes may be grouped into five major functional categories (Table 3). Processes related to energy metabolism were highly represented, including the generation of precursor metabolites and energy (*p*-value_adj_ = 0.01), cellular respiration (*p*-value_adj_ = 0.02), acetyl-CoA biosynthetic process from pyruvate (*p*-value_adj_ < 0.01), tricarboxylic acid cycle (*p*-value_adj_ = 0.04), and isocitrate metabolic process (*p*-value_adj_ = 0.04). Within the category of compound transport and signaling, nitrogen compound transport (*p*-value_adj_ = 0.02) and regulation of calcium ion transmembrane transporter (*p*-value_adj_ < 0.01) were enriched. The complex assembly category was represented by mitochondrial respiratory chain complex assembly (*p*-value_adj_ < 0.01), while the structural category included protein folding (*p*-value_adj_ = 0.03). Finally, within signaling, regulation of muscle system process (*p*-value_adj_ = 0.04) was identified.

**Table 3:** Enriched biological processes of the exclusive protein in the skeletal muscle of calves from slow-release nitrogen (SRN) treatment.

ID	Description	*p*-value_adj_	Protein Symbols
***Energy metabolism***			
GO:0006091	Generation of precursor metabolites and energy	0.01	ATP5F1D, ETFB, IDH1, IDH3G, NDUFA8, NDUFA13, NDUFS4, PDHA1 (mitochondrial)
GO:0045333	Cellular respiration	0.02	ATP5F1D, IDH1, IDH3G, NDUFA8, NDUFS4, PDHA1 (mitochondrial)
GO:0006086	Acetyl-CoA biosynthetic process from pyruvate	< 0.01	PDHA, PDHA1, PDHX (mitochondrial)
GO:0006099	Tricarboxylic acid cycle	0.04	IDH1, IDH3G, PDH1 (mitochondrial)
GO:0006102	Isocitrate metabolic process	0.04	IDH1, IDH3G (mitochondrial)
***Compound transport signaling***			
GO:0071705	Nitrogen compound transport	0.02	AIFM1, NDUFA13, PAM16 (mitochondrial), CAV1, LMNA, GCC2, SAR1A, SAR1B, VPS51, RPL11 (structural/organelle-associated), GDI1, GDI2, HNRNPA2B1, HSPA5, YWHAB (regulatory/signaling)
GO:1901019	Regulation of calcium ion transmembrane transporter	< 0.01	ATP1B1, CACNA2D1, TRDN (transporter), CAV1, CAV3 (structural), DHRS7C, FKBP1A (regulatory/chaperone)
***Complex assembly***			
GO:0032981	Mitochondrial respiratory chain complex assembly	< 0.01	NDUFA8, NDUFA11, NDUFA12, NDUFA13, NDUFB7, NDUFB10, NDUFS4(mitochondrial)
***Structural***			
GO:0006457	Protein folding	0.03	CCT4, FKBP1A, HSPA5, HSPA6, PPIA, STIP1(chaperones)
***Signaling***			
GO:0090257	Regulation of muscle system process	0.04	ATP1A1, ATP1B1, CAV1, CAV3, LMNA (membrane proteins)

### Function of the differentially abundant proteins (DAPs)

Enrichment analysis of all the 27 DAPs identified 13 proteins associated with four significant biological processes (*p*-value_adj_ ≤ 0.05; Fig 3). These biological processes include ATP metabolic process (ALDOA, ATP5A1, ATP5B, ATP5C1, PKM, TPI1), glucose metabolic process (DLAT, MDH2, PGM1, PKM, TPI1), tricarboxylic acid cycle (CS, DLAT, MDH2), and sarcomere organization (FLNC, MYBPC1, MYOM2) (Fig 3), reflecting key pathways in energy metabolism and muscle structure.

## Discussion

Gestation is a challenging period of animals’ life, since it represents the increase in maternal energy demands to ensure the proper development of the fetus and the necessary supply of nutrients [6]. Additionally, it involves preparing maternal metabolism for parturition and lactation. Nitrogen deficiency, commonly observed in tropical pastures during the dry season, may trigger mechanisms of maternal lean tissue mobilization as an attempt to provide glucogenic precursor to support fetal development [26]. However, protein supplementation for pregnant beef cows under grazing conditions may attenuate this tissue mobilization [7,8] in addition to enhance glucose availably for fetal development [8].

Preliminary results, show that slow-release nitrogen enriched diets during gestation improved maternal forage utilization and positively affected offspring’s performance [27]. Specifically, the pregnant cows fed slow-release nitrogen diets during late gestation presented greater dry matter intake, greater average daily gain, and an increased body condition score compared to the control group [27]. Moreover, maternal effects may have contributed to increase calves weaning weights [27]. Based on this evidence, we hypothesize that supplementing slow-release nitrogen sources during bovine development may enhance skeletal muscle growth in the offspring by modulating transcriptomic and proteomic profiles.

No differences were observed at the transcriptome level. However, the proteomic profile of the skeletal muscle of the progeny was altered as a result of maternal slow-release nitrogen diet. The enriched biological process in the skeletal muscle of calves from SRN group is associated with ATP metabolic process and glucose metabolism. The enrichment was evidenced through the greater abundance of the DAPs: phosphoglucomutase 1 (PGM1), aldolase (ALDOA), triosephosphate isomerase (TPI1), and pyruvate kinase (PKM).

Although PGM1 plays a role in both glycolysis and glycogenesis processes [28], the greater abundance of other proteins downstream the glycolysis pathway suggest that the storage of muscle glycogen is directed toward the synthesis of pyruvate. This assumption is supported by the increased abundance of PKM, which catalyzes the irreversible conversion of phosphoenolpyruvate into pyruvate, contributing to ATP formation [29]. Consistently, a reduced abundance of the DAP citrate synthase (CS) may indicate lower concentration of citrate, which is recognized to inhibit pyruvate synthesis mediated by PKM [30]. Interesting, both ALDOA and TPI1 regulate the abundance of the metabolites dihydroxyacetone phosphate (DHAP) and glyceraldehyde 3-phosphate (GAP). Specifically, ALDOA cleave fructose- 1,6-biphosphate into DHAP and GAP, while TPI1 catalyzes the conversion of this metabolites [31]. The formation of DHAP contributes to lipid metabolism through the conversion into glycerol, which is a crucial component for triglyceride synthesis. Despite the formation of DHAP, proteins directly involved in lipid synthesis were not differentially abundant in our study. Additionally, the reduced abundance of CS in the skeletal muscle of calves from the SRN treatment may indicate lower citrate availability for cytosolic transport, potentially limiting de novo fatty acid synthesis. In suckling calves, nutrient partitioning favors energy generation to sustain rapid muscle accretion rather than lipid deposition, with lipogenesis becoming more pronounced after puberty [32]. Taken together, these findings suggest that our treatment may have promoted the conversion of DHAP into GAP. Consequently, the oxidation of GAP through the energy-generation phase of glycolysis, accompanied by the greater abundance of PKM may have resulted in greater synthesis of ATP, NADH and pyruvate in the calves born from cows which received a slow-release nitrogen enriched diets during gestation.

Once pyruvate is formed it may follow the oxidative decarboxylation through the regulation of the pyruvate dehydrogenase complex (PDC). This complex is characterized by its three catalytic subunits and the final formation of acetyl-CoA, CO_2_ and NADH [33]. Proteins (PDHA, PDHA1, PDHX) that are components of the E1 subunit of the PDC were found exclusively in the skeletal muscle of calves from SRN treatment. However, lower abundance of the protein component of E2 subunit (DLAT) was reported in the skeletal muscle of the calves from the same treatment (SRN). These divergences may be associated with the levels of mitochondrial citrate. Higher concentrations of citrate, produced in the skeletal muscle of calves from CON treatment, might inhibit E1 subunit of the PDC complex [30]. Thus, the increased abundance of DLAT (E2 subunit) in CON animal, could potentially act as a compensatory mechanism to ensure the continued production of Acetyl-CoA, maintaining the positive feedback for citrate formation. While assumptions can be made regarding the abundance of proteins associated with the subunits of PDC, it is important to note that the activity of this complex is mediated by post-translational modifications. In brief, the PDC can be deactivated through phosphorylation mediated by kinases, whereas activation is mediated by phosphatases [34]. Therefore, as we did not assess the phosphoproteome, we cannot conclusively state whether our experimental treatment is influencing the activity of PDC. Future studies incorporating phosphoproteomics and metabolomics, within a systems biology approach, could provide valuable insights and help to comprehensively understand the metabolic consequences of maternal slow-release nitrogen supplementation.

Besides the contribution of glycolysis for the generation of NADH, crucial process of the citric acid cycle contributes to the enhancement in NADH production. For instance, the conversion of malate into oxaloacetate mediated by the mitochondrial malate dehydrogenase (MDH2), and the conversion of isocitrate into α-ketoglutarate (α-KG) mediated by isocitrate dehydrogenase (IDH) are reactions that contributes to NADH formation [29]. In the current study slow-release nitrogen diets during gestation promoted greater abundance of MDH2, in addition to the synthesis of subunit γ of IDH in the skeletal muscle of the offspring. All provided reducing equivalents from these reactions appear to be directed toward the electron transport chain (ETC) in the inner membrane of mitochondria to form ATP.

Exclusive proteins associated with the complex I of the ETC were found in the skeletal muscle of calves from SRN treatment such as NDUFA8, NDUFA11, NDUFA12, NDUFA13, NDUFB7, NDUFB10, and NDUFS4. The complex I of the ETC is the largest component of the mitochondrial oxidative phosphorylation system and functions by oxidizing NADH [35]. During this process, complex I transfer electron from NADH to ubiquinone, forming ubiquinol [35]. This contribution is essential for creating the proton gradient necessary for ATP synthesis [35,36]. Moreover, among the ETC complexes, complex I and III are known to be greater producers of reactive oxygen species (ROS), which are important molecules in several signaling pathways, including apoptosis [35]. It is important to mention that slow-release nitrogen enriched diets promoted greater abundance of IDH1 in the skeletal muscle of the calves. This enzyme reaction generates α-KG and NADPH in cytosol [37]. While the role of cytosolic α-KG remains unclear, NADPH may play an essential role maintaining cellular redox balance and antioxidant defense [38]. Additionally, greater abundance of Voltage-dependent anion-selective channel protein 3 (VDAC3) in the skeletal muscle of calves from SRN group could be linked to elevated ROS production from the ETC. VDCA3 functions as a sensor of mitochondrial ROS levels [39]. Knocking out VDCA3 has been shown to lead to the accumulation in mitochondrial free radicals, resulting in oxidative stress [40]. Therefore, VDCA3 is indispensable to counterbalance ROS production from complex I [40]. Thus, the increased activity of complex I in ROS production is likely controlled by the synthesis of NADPH and VDCA3 to maintain balance in the skeletal muscle of calves from SRN treatment.

In addition to the observed impact on complex I of the ETC, our experimental treatment with slow-release nitrogen during gestation, also revealed a higher abundance of proteins associated with complex V (ATP5A1, ATP5C1, and ATP5B) in the skeletal muscle of the calves. Complex V, also known as ATP synthase, plays a crucial role in ATP synthesis by utilizing the energy generated from the electrochemical gradient established by the ETC [36]. This complex catalyzes the phosphorylation of ADP into ATP during cellular respiration [36]. The enhancement in the abundance of proteins linked to complex V suggests an improvement in ATP synthesis efficiency, indicating a positive impact of the experimental treatment on the bioenergetic process in the skeletal muscle of calves. These findings highlight the complexity of cellular responses induced by our slow-release nitrogen enriched diets, demonstrating not only the enhancement in electron transfer process but also the improvement in ATP production.

Due to its contractile properties, skeletal muscle requires a large number of mitochondria to provide ATP. In addition to mitochondria functions, the skeletal muscle of calves from SRN group exhibited enriched biological process associated with sarcomere organization, involving the DAPs: myomesin 2 (MYOM2), myosin-binding protein C 1 (MYBPC1), and filamin C (FLNC). The sarcomere is divided into specialized distinct compartments with the Z-disc defining sarcomere boundaries and anchoring the thin filaments (actin) [41]. The isoform C of filamin (FLNC) is localized in the Z-disc and links actin to numerous binding partners, including receptors, channels, intracellular signaling molecules, and transcription factors [42]. For instance, FLNC phosphorylation prevents FLNC proteolysis mediated by calpains [43]. In a meat industry scenario, this situation would impair beef tenderization. However, since the phosphoproteome was not assessed in the current study, assumptions on how our treatment would affect meat tenderization need to be further clarified.

The central part of the sarcomere, called M-band, is where the thick filaments (myosin) interconnect and run antiparallel to both sides of the sarcomere [41]. MYOM2 is localized in M band, and is present in the majority of fast muscle fibers [41]. In addition, it was reported that myomesin proteins may mediate the binding of myofibrils with muscle-type creatine kinase (CKM) [44]. CKM allows ATP regeneration by transferring phosphate groups from phosphocreatine to ADP, thus enabling the continuous processes of muscle contraction [28]. Besides myomesin, another study reported that MYBPC1 also function as a binding partner of CKM, providing rapid ATP regeneration [45]. Hence, these results, associated with the greater abundance of CKM in the skeletal muscle of calves from SRN group, suggest a potential mechanism for efficient ATP turnover in muscle contraction and relaxation process.

## Conclusions

**Fig 4:**
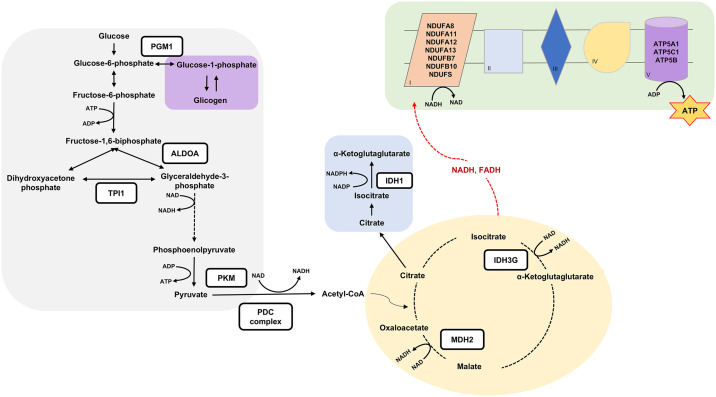
Summarized pathways influenced by the maternal slow-release nitrogen diets during gestation. Proteins inside the black squares were more abundant or exclusive expressed in the skeletal muscle of the calves from SRN treatment. Glycolysis/glycogenesis is represented in gray, glycogenolysis in purple, citrate cycle in yellow, cytoplasmic conversion of citrate into α-ketoglutarate in blue, and electron transport chain in green.

In summary, findings from the current study indicate that slow-release nitrogen-enriched diets during late gestation had positive effects on the energy metabolism of offspring’s skeletal muscle (Fig 4). The greater abundance of proteins involved in glycolysis, the citric acid cycle, complex I and V of the electron transport chain, and structural proteins suggest improvements in bioenergetic efficiency, ATP synthesis, and structural organization of the skeletal muscle.

## Supporting information

S1 Fig Venn diagram of the proteins identified. Number of proteins identified in each treatment (exclusive), the intercept containing the number of proteins common in both treatments. SRN = Slow-released Nitrogen; CON = Control.
(TIF)

S2 Fig Multidimensional scaling (MDS) plot showing the relative similarities of the samples of the treatments SRN (slow-release nitrogen) and CON (control). (TIFF)

S3 Fig Heatmap containing the 100 most variables genes. (TIFF)

S4 Fig Mean-difference plot showing the log2-fold change (logFC) and average abundance of each differentially expressed (DE) genes between treatments. Black dots represent the non-differentially expressed genes.
(TIFF)

S1 Table Summary of the number of trimmed reads, and the output counting file containing unmapped, multimapping, noFeature and Ambiguous reads. SRN = Slow-release nitrogen; CON = Control; ^1^Mapped = Trimmed reads – Unmapped – Multimapping – noFeature - Ambiguous.
(DOCX)
