# Supplementary figures and images for: Maternal slow-release nitrogen diets during late gestation optimize the energy metabolism in calves’ skeletal muscle
Source: PLoS One. 2026 Jan 30;21(1):e0338860. doi: 10.1371/journal.pone.0338860 (PMC12858008; doi:10.1371/journal.pone.0338860)

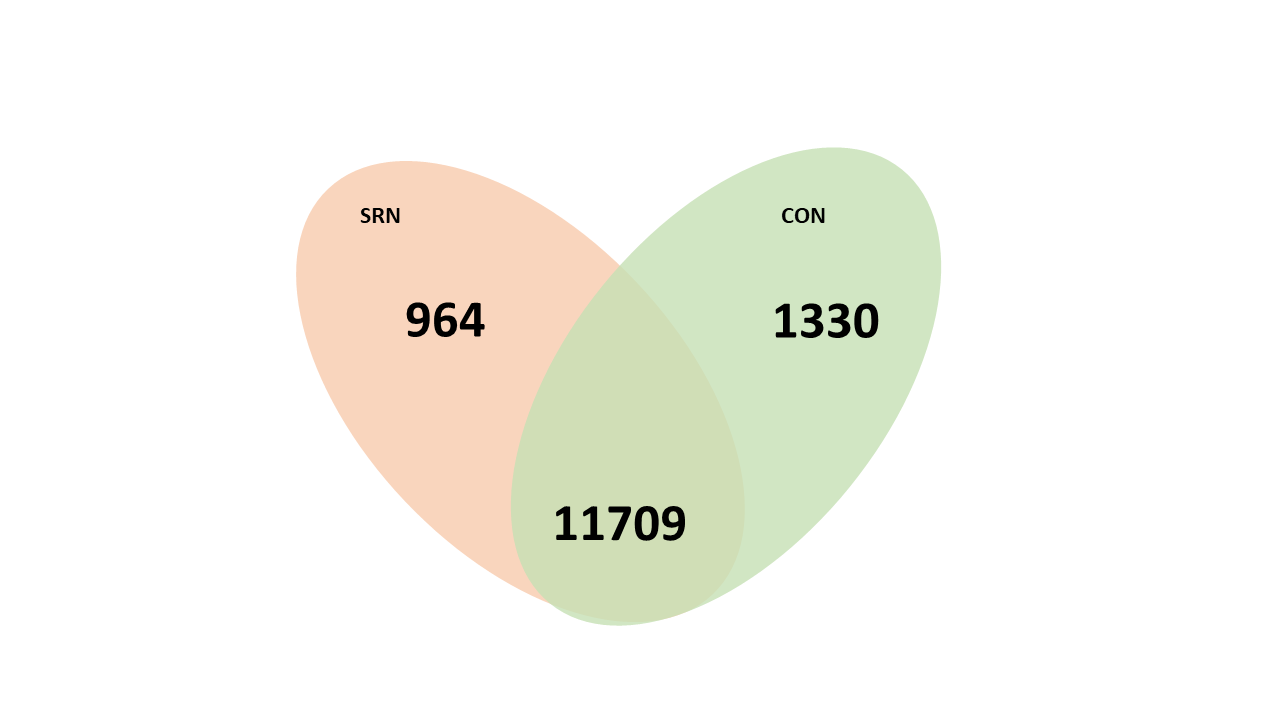

Supplement: S1 Fig — Number of proteins identified in each treatment (exclusive), the intercept containing the number of proteins common in both treatments. SRN = Slow-released Nitrogen; CON = Control. (TIF) [file pone.0338860.s001.tif]

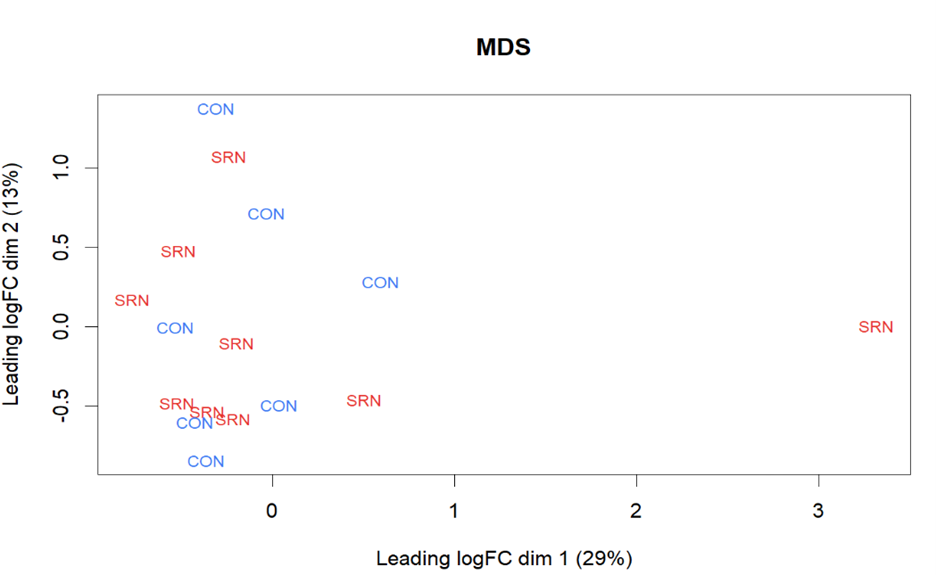

Supplement: S2 Fig — (TIFF) [file pone.0338860.s002.tiff]

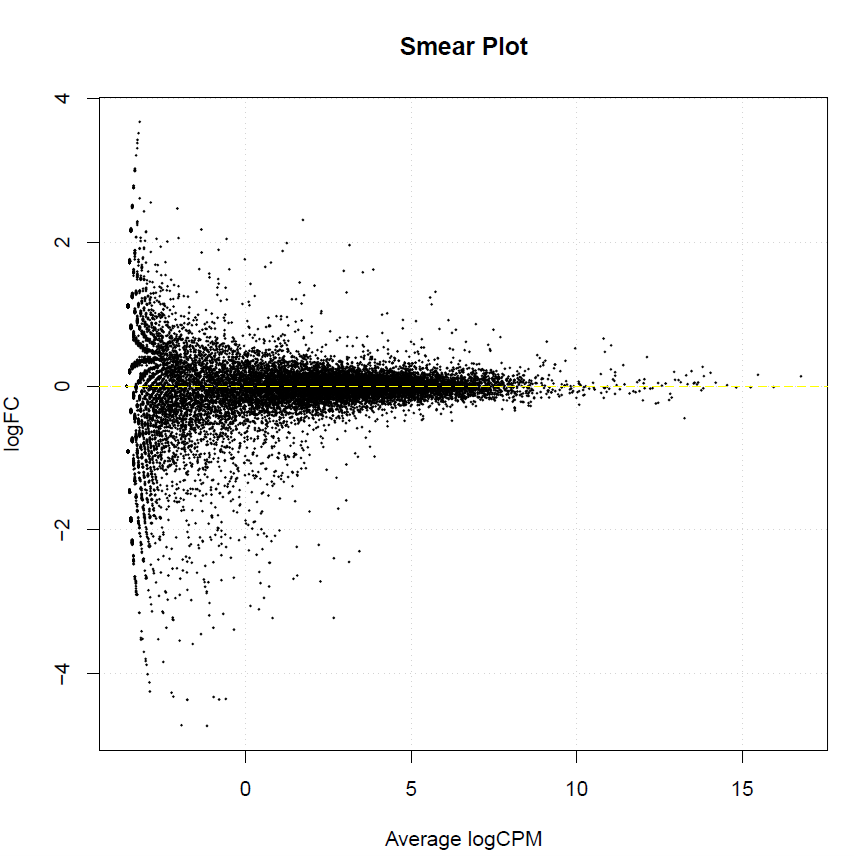

Supplement: S4 Fig — Black dots represent the non-differentially expressed genes. (TIFF) [file pone.0338860.s004.tiff]
