# Supplementary material for: Maternal slow-release nitrogen diets during late gestation optimize the energy metabolism in calves’ skeletal muscle
Source: PLoS One. 2026 Jan 30;21(1):e0338860. doi: 10.1371/journal.pone.0338860 (PMC12858008; doi:10.1371/journal.pone.0338860)
Supplement: S1 Table — SRN = Slow-release nitrogen; CON = Control; 1Mapped = Trimmed reads – Unmapped – Multimapping – noFeature - Ambiguous. (DOCX) [file pone.0338860.s005.docx]

| **Sample** | **Treatment** | **Trimmed reads** | **Unmapped** | **Multimapping** | **noFeature** | **Ambiguous** | **Mapped^1^** | **%Mapped** |
| --- | --- | --- | --- | --- | --- | --- | --- | --- |
| **101** | **SRN** | 24119255 | 294751 | 290315 | 2467403 | 350956 | 20715830 | 85.89 |
| **103** |  | 24054627 | 358912 | 327519 | 2406444 | 263375 | 20698377 | 86.05 |
| **106** |  | 24088351 | 344051 | 321843 | 2598727 | 258882 | 20564848 | 85.37 |
| **108** |  | 24109890 | 299477 | 311108 | 2605727 | 271233 | 20622345 | 85.53 |
| **111** |  | 24018940 | 334134 | 308748 | 2538911 | 401273 | 20435874 | 85.08 |
| **115** |  | 24107314 | 273425 | 301920 | 2236509 | 363864 | 20931596 | 86.83 |
| **118** |  | 24009843 | 320882 | 282889 | 2810801 | 377195 | 20218076 | 84.21 |
| **122** |  | 24003117 | 334870 | 351076 | 3072091 | 224160 | 20020920 | 83.41 |
| **128** |  | 24109126 | 326173 | 322529 | 2770944 | 258828 | 20430652 | 84.74 |
| **102** | **CON** | 24104953 | 447451 | 282978 | 2970881 | 379686 | 20023957 | 83.07 |
| **104** |  | 24043443 | 413506 | 304427 | 2771452 | 239871 | 20314187 | 84.49 |
| **105** |  | 24105418 | 342381 | 332942 | 2746466 | 275850 | 20407779 | 84.66 |
| **110** |  | 24094067 | 314231 | 328743 | 2317026 | 337967 | 20796100 | 86.31 |
| **112** |  | 24111556 | 329650 | 336563 | 2436492 | 241172 | 20767679 | 86.13 |
| **124** |  | 24080741 | 394601 | 299821 | 3255102 | 455315 | 19675902 | 81.71 |
| **126** |  | 24031033 | 378910 | 333282 | 3094297 | 185857 | 20038687 | 83.39 |
